# Hyperleptinemia in children with autosomal recessive spinal muscular atrophy type I-III

**DOI:** 10.1371/journal.pone.0173144

**Published:** 2017-03-09

**Authors:** Heike Kölbel, Berthold P. Hauffa, Stefan A. Wudy, Anastasios Bouikidis, Adela Della Marina, Ulrike Schara

**Affiliations:** 1 Department of Neuropediatrics, Developmental Neurology and Social Pediatrics, Children’s Hospital 1, University of Duisburg-Essen, Essen, Germany; 2 Department of Pediatric Endocrinology, Children’s Hospital 2, University of Duisburg-Essen, Essen, Germany; 3 Steroid Research and Mass Spectrometry Unit, Division of Pediatric Endocrinology and Diabetology, Center of Child and Adolescent Medicine, Justus Liebig University, Giessen, Germany; 4 Department of Pediatric Pulmonology, Children’s Hospital 3, University of Duisburg-Essen, Essen, Germany; University of Edinburgh, UNITED KINGDOM

## Abstract

**Background:**

Autosomal-recessive proximal spinal muscular atrophies (SMA) are disorders characterized by a ubiquitous deficiency of the survival of motor neuron protein that leads to a multisystemic disorder, which mostly affects alpha motor neurons. Disease progression is clinically associated with failure to thrive or weight loss, mainly caused by chewing and swallowing difficulties. Although pancreatic involvement has been described in animal models, systematic endocrinological evaluation of the energy metabolism in humans is lacking.

**Methods:**

In 43 patients with SMA type I-III (8 type I; 22 type II; 13 type III), aged 0.6–21.8 years, auxological parameters, pubertal stage, motor function (Motor Function Measurement 32 –MFM32) as well as levels of leptin, insulin glucose, hemoglobin A1c, Homeostasis Model Assessment index and an urinary steroid profile were determined.

**Results:**

Hyperleptinemia was found in 15/35 (43%) of our patients; 9/15 (60%) of the hyperleptinemic patients were underweight, whereas 1/15 (7%) was obese. Hyperleptinemia was associated with SMA type (p = 0.018). There was a significant association with decreased motor function (MFM32 total score in hyperleptinemia 28.5%, in normoleptinemia 54.7% p = 0.008, OR 0.969; 95%-CI: 0.946–0.992). In addition, a higher occurrence of hirsutism, premature pubarche and a higher variability of the urinary steroid pattern were found.

**Conclusion:**

Hyperleptinemia is highly prevalent in underweight children with SMA and is associated with disease severity and decreased motor function. Neuronal degradation of hypothalamic cells or an increase in fat content by muscle remodeling could be the cause of hyperleptinemia.

## Introduction

Autosomal-recessive proximal spinal muscular atrophies (SMA) are monogenetic progressive disorders characterized by a ubiquitous deficiency of the survival of motor neuron (SMN) protein, leading to a multisystemic disorder which, for unexplained reasons, appears to affect mostly alpha motor neurons [1]. SMN protein interacts with more than 100 proteins so that a lower SMN protein level should have significant downstream molecular consequences which affect a range of different target proteins und pathways [2].

SMA is the most common genetic cause of infant mortality and seems to be present in all populations. The SMA type is defined by the time of onset of symptoms and highest achieved motor milestone. Until now, the disorder has been untreatable, and management relies on supportive care to address disease complications and maximize clinical and motor functions [3]. Recent studies have shown that severe SMA type I can result in cardiac, vascular, brain and sensory nerve involvement [4–6] including thalamic lesions in SMA type I patients [7]. In an *Smn−/−/n−/2* mouse model could be shown that low SMN protein level disrupt proliferation and neurogenesis and plays an important role in brain development, and SMN protein deficiency resulted in defective hippocampal development [8].

One of the most important clinical signs for disease progression is the failure to thrive or weight loss, especially in SMA type I patients [9]. This is thought to be caused by chewing and swallowing difficulties as a result of muscle weakness and respiratory distress [10]. In contrast, high-functioning SMA type II patients are at risk of becoming overweight, and they have a higher relative fat mass [11].

With regard to energy metabolism, abnormal fatty acid metabolism is the most common metabolic defect reported in severe and younger SMA type I and II patients, mild to moderate dicarboxylic aciduria is consistently found in SMA types I and II patients, which is very similar to mitochondrial b oxidation abnormalities. Further evidence of fatty acid oxidation disorders is based on the autopsy samples of some infants with severe SMA who showed fatty vacuolization of the liver [12]. The role of SMN protein in the development and function of liver in mice was demonstrated by a study in which a mutation in the exon 7 of murine Smn directed to liver led to liver failure and late embryonic lethality of transgenic mice [13].

A case report presented a 29-year-old man with genetically confirmed spinal muscular atrophy type II with new onset of diabetes mellitus type 2 and diabetic ketoacidosis [14]. Metabolic defects in an intermediate mouse model (SMA^2B/−^) were characterized by fasting hyperglycemia, glucose intolerance, hypersensitivity to insulin, and hyperglucagonemia. Pathological defects were identified by loss of insulin-producing β cells and a corresponding increase in the number of the glucagon-producing α cells in pancreatic islets of mice and human specimens. Based on the observation that the pancreatic pathology and fasting hyperglycemia occurred before the onset of SMA symptoms, the authors suggested that the pancreatic phenotype is independent of the neuronal SMA phenotype and is rather a direct consequence of SMN deficiency [15]. Further, there appears to be an influence of maternal diet on the survival and motor phenotype of transgenic mice (SMNDelta7 SMA mice (SMN2(+/+); SMNDelta7(+/+); mSmn(-/-)) [16]. Clinical studies of hormonal disturbances of energy metabolism and linkage to disease severity in humans are lacking. In another motor neuron disease, amyotrophic lateral sclerosis (ALS), hypermetabolism is a well-known factor for disease progression. As a consequence, a high energy diet has been recommended early in ALS disease course [17]. To study the influence of hypermetabolism, a mouse model for ALS combined with leptin deficiency (SOD1 mice +*ob/ob*) was created. Data showed that decreased serum leptin levels reduced weight loss and energy expenditure as well as the degeneration of motor neurons. This led to an improved motor function and increased the longevity of the SOD1 mice [18].

To further elucidate the endocrine and metabolic consequences of SMA, we conducted a prospective study by including an endocrinological investigation into our standard of care protocol [3] in patients with SMA type I-III. Here we present the first data concerning energy metabolism.

## Patients and methods

### Study setting

The study was conducted at the Department of Neuropediatrics of the University Children’s Hospital, University Duisburg-Essen, in a tertiary care setting. 6400 patients visit the Department of Neuropediatrics per year; 1400 of them have neuromuscular diseases. Sixty patients with genetically confirmed SMA type I-III participate in our standard of care program on a regular basis; 43 out of these 60 SMA patients (8 SMA type I; 22 SMA type II; 13 SMA type III) were recruited for this study. Patient age ranged from 6 months to 21 years (mean age 8.63 years) with a balanced sex ratio (21 female/22 male patients).

### Ethics

Consent of ethical review committee (ERC): The patients or their legal representatives provided written informed consent approved by the central institutional ethical review board (12-5015-Bo) at the enrolling site.

### Auxological methods (human physical growth parameters)

In non-ambulant patients length was measured in supine position with a tape. In ambulant patients height was recorded with a wall-mounted stadiometer (Ulmer Stadiometer, Elchingen, Germany). Height/length was recorded to the nearest 0.1 cm. In non-ambulant patients weight was measured with a wheelchair scale for medical use (seca 665, Hamburg, Germany) and in ambulant patients with a medical scale (seca 319, Hamburg, Germany) with a precision of 100 g. BMI, defined as body mass (kg) divided by the square of the body height (m^2^), was classified according to the percentile (P) range as follows: < 3. P = extreme underweight; 3.≤ 10. P = underweight; 10.-90. P = normal weight; ≥ 90. P = overweight; ≥ 97. P = obesity. Waist circumference (WC) was measured in supine position with a tape approximately 3 cm above the umbilicus and recorded to the nearest 0.5 cm. Hip circumference (HC) was measured by tape in supine position around the largest prominence of the pelvis and recorded to the nearest 0.5 cm. Waist-to-Hip Ratio (WHR) was calculated by dividing waist and hip circumference.

Height/length was transformed into height SD score (HtSDS) using the data of Reinken [16]. In the age range < 18 years, weight and BMI were transformed into their respective percentiles or SD scores (SDS) based on the reference data of Kromeyer-Hauschild [19]. SD scores for WC, HC and WHR were calculated using data from the Dutch population [20]. All SD scores of the above-mentioned adiposity indices were calculated using the LMS method [21].

Pubertal development was described according to Marshall and Tanner [22, 23]; abundance of body hair was described using the Ferriman-Gallway score [24]. Body hair is rated in nine different skin areas with a score of 0 to 4; areas with no hair are scored 0. If the total score was ≥ 6, hirsutism was present [25].

### Neuromuscular function testing

The Motor Function Measurement Scales 32 (MFM32) is a widely recognized method to determine the motor function in patients with neuromuscular diseases [26]. The scale comprises 32 items in three dimensions: standing position and transfers (D1), axial and proximal motor function (D2), distal motor function (D3). The items are tested in supine, seated or standing positions. The items are numbered from 1–32 and arranged in a logical order. Several major criteria were primarily used to distinguish between the patient's usual ability to walk, run and jump (for D1), his/her usual ability to sit upright, perform activities involving proximal motor actions and hold his/her head up (for D2) and his/her usual ability to handle objects (for D3). The total score is the sum of the 32 items scores. We applied this method according to the guidelines. The test was performed in all patients by the same certified pediatrician. Two children aged < 12 months were assessed with the Children’s Hospital of Philadelphia Infant Test of Neuromuscular Development (CHOP Intend); these results are not comparable with those of the MFM32, as these are two completely different tests in terms of motoric demand to the children; thus, the results were not included into the statistical analysis.

### Laboratory methods

Leptin (ng/ml) was measured in serum using a commercial kit (hLeptin SENSITIV ELISA^®^, Mediagnost, Reutlingen, Germany). Serum leptin concentrations depend on the following factors: patient’s sex, age, BMI and pubertal status. To make leptin concentrations comparable between disease groups and levels of motor function, the influence of the above factors must be removed. To do so, we converted the original values into SDS using an algorithm developed by Blum [27].

Serum glucose (mmol/l) concentrations were determined enzymatically by the hexokinase method (ADVIA Clinical Chemistry (Siemens/Erlangen; normal fasting values 3.3–5.5 mmol/l). Plasma fasting insulin concentrations (μU/ml) (last meal < 6 hours) were measured by RIA (Immulite 2000 Xpi/Siemens/Erlangen; normal values 3.6–29.1 μU/ml).

The Homeostasis Model Assessment (HOMA) Insulin Resistance (IR) index was calculated for all participants as an indicator or IR (fasting insulin μU/ml x fasting glucose mmol/l / 22.5) according to Matthews [28].

Hemoglobin A1c (HbA1c) was measured by chromatography and expressed as the ratio of glycosylated hemoglobin A1c to total hemoglobin in % (TOSOH Bioscience/Erlangen, normal values 4.0–6.05%).

Urinary steroid profile: The pattern of urinary steroid excretion was determined by gas chromatography and mass spectrometry (GC-MS) at the pediatric steroid research unit of the University Children’s Hospital, University of Giessen [29].

### Statistical analysis

Categorical and ordinal characteristics have been described by specifying absolute and relative occurrence and metric characteristics based on the mean standard deviation, median, minimum and maximum. Whether the median of a metric characteristic equals zero was tested with the sign test. Odds Ratios were used to quantify the relation between hyperleptinemia and the scores of the motor function test. Odds ratio (OR) greater or smaller than 1 were labeled as “associated with“. Confidence Interval (CI) was estimated where a 95% confidence interval reflects a significance level of 0.05. The influence of leptin SDS and SMA type on the total score of MFM32 was evaluated by linear regression. The same method was used to analyse whether leptin SDS is influenced by SMA type or sex. Further, we used the Fisher's exact test, the analysis of variance (ANOVA) and the Mann-Whitney U-test. All statistical tests were two-sided with a significance level of 0.05. For data processing and statistical analysis Stata / IC 13.1 for *Windows* was used.

## Results

### Energy metabolism

#### Leptin

Of the 35 patients in whom leptin was measured, 20/35 (57%) had normoleptinemia and 15/35 (43%) were hyperleptinemic. An association of sex (p = 0.763) with leptin SDS was not found. SMA type and leptin SDS were strongly linked (p = 0.006) (Table 1).

**Table 1:** Association between leptin SDS and SMA type.

	estimatedcoeff. [%]	95%-CI	p-value (t-test)
Boys vs. girls	-0.23	-1.74–1.29	0.763
SMA type II vs. I	-1.95	-4.20–0.29	0.086
**SMA type III vs. I**	**-3.39**	**-5.74 - -1.05**	**0.006**
constant	4.56	2.62–6.50	<0.001

Vs. = versus, CI = Confidence Interval, coeff. = Coefficient, SMA = spinal muscular atrophy, constant = estimated mean average of leptin SDS from girls with SMA type 1

Table 1: The analysis of the association between leptin SDS and SMA type by linear regression showed that the SMA type had a significant influence on leptin-SDS levels (SMA-global test, p = 0.006) according to the great difference of leptin SDS between SMA type I and III patients. There was no association between sex and leptin SDS (p = 0.763).

We then assessed the association of body fat and its distribution (BMI, weight, WC and WHR) with leptin SDS. Of the patients with hyperleptinemia, 9/15 (60%) were underweight, 5/15 (33%) had normal weight and 1/15 (7%) were obese, according to BMI percentiles. The preponderance of underweight subjects among hyperleptinemic patients was significant (p = 0.032; OR: 6.0 (1.1–32.0). Weight, WC and WHR were not correlated with hyperleptinemia.

**Fig 1:**
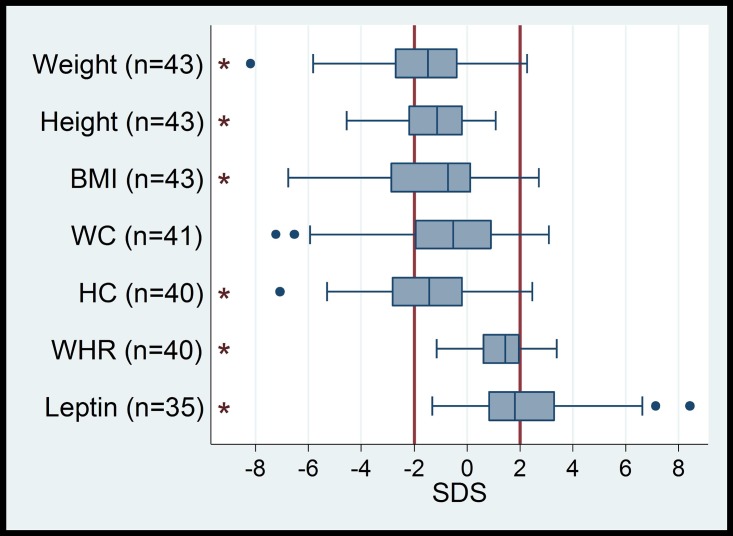
The relation between motor function and leptin SDS in terms of SMA type, showing that the lower the overall motor function, the higher was the risk for elevated leptin levels. Vertical lines in bold at -2 SD and +2 SD indicate the reference range for leptin SDS. **As a consequence, lower motor function is linked to high leptin-SDS independent of SMA type**.

Further, we examined a possible association of leptin with motor function, measured by MFM32, a surrogate marker for the severity of the disease (Tables 2 and 3 and Fig 1).

**Table 2:** Influence of leptin SDS and SMA type on motor function.

MFM32		estimatedcoeff. [95%-CI]	p-value(t-Test)	SMA—globaltest (F-Test)
D1: standing	Leptin SD	0.2 (-1.3–1.6)	0.799	
position	SMA II vs. I	3.2 (-1.6–8.1)	0.183	
and transfers	**SMA III vs. I**	**56.6 (33.0–80.1)**	**<0.001**	**<0.001**
	constant	-0.3 (-8.1–7.5)	0.935	
D2: axial and	**Leptin SD**	**-3.6 (-7.5–0.3)**	**0.066**	
proximal	SMA II vs. I	28.7 (8.3–49.1)	0.007	
motor	**SMA III vs. I**	**67.8 (43.8–91.8)**	**<0.001**	**<0.001**
function	constant	28.2 (3.0–53.4)	0.029	
D3: distal	Leptin SD	-2.7 (-6.8–1.4)	0.184	
motor	SMA II vs. I	26.8 (-9.1–62.7)	0.137	
function	**SMA III vs. I**	**67.4 (30.0–104.8)**	**0.001**	**<0.001**
	constant	31.0 (-9.6–71.6)	0.129	
Total	Leptin SD	-1.9 (-4.2–0.3)	0.094	
score	SMA II vs. I	17.8 (2.4–33.3)	0.025	
	**SMA III vs. I**	**62.7 (42.1–83.3)**	**<0.001**	**<0.001**
	constant	17.4 (-0.7–35.5)	0.060	

MFM32 = Motor Function Measurement32, vs. = versus; SD = Standard Deviation, coeff. = Coefficient; CI = Confidence Interval; SMA = spinal muscular atrophy

**Table 3:** Association between leptin SDS and motor function.

Leptin—MFM32 D1: standing position and transfers (%)						
Leptin	n	mean	SD	median	min-max	p-value
						Odds Ratio (95%-CI)
norm	14	33.6	34.1	19.2	0.0–84.6	
> norm	20	10.9	24.9	2.6	0.0–89.0	p = 0.034
total	34	20.2	30.7	5.1	0.0–89.0	OR: 0.976 (0.955–0.998)
Leptin—MFM32 D2: axial and proximal motor function (%)						
Leptin	n	mean	SD	median	min-max	p-value
						Odds Ratio (95%-CI)
norm	14	72.2	27.4	77.8	30.5–100.0	
> norm	20	47.5	32.5	50.0	0.0–100.0	p = 0.029
total	34	57.7	32.5	55.5	0.0–100.0	OR: 0.977 (0.957–0.998)
Leptin—MFM32 D3: distal motor function (%)						
Leptin	n	mean	SD	median	min-max	p-value
						Odds Ratio (95%-CI)
norm	14	72.1	25.1	66.7	33.3–100.0	
> norm	20	54.0	34.7	57.1	0.0–100.0	p = 0.103
total	34	61.4	32.0	59.5	0.0–100.0	OR: 0.982 (0.962–1.004)
Leptin—MFM32: Total score (%)						
Leptin	n	mean	SD	median	min-max	p-value
						Odds Ratio (95%-CI)
norm	14	56.3	28.4	51.0	24.0–93.8	
> norm	20	33.9	25.9	34.4	0.0–96.0	p = 0.026
total	34	43.1	28.8	34.8	0.0–96.0	OR: 0.973 (0.951–0.997)

n = numbers, SD = Standard Deviation, min = minimun, max = maximum, OR = Odds ratio; CI = Confidence Interval; MFM32 = Motor Function Measurement32

Table 2: Influence of leptin SDS and SMA type on the 3 dimensions and total score of MFM32 evaluated by linear regression (n = 34). The analysis confirms a significant influence of SMA type on motor function and indicates an association of leptin with motor function in subtest D2 (p = 0.066). This verifies that motoric function of our patients match SMA type, indicating that the majority our patients have not shown a relevant motoric decline so far.

Table 3: Association between leptin SD scores and subtests and total scores of the MFM32 motor function test. Hyperleptinemia (>+2 SDS) is associated with increased motor function bar the distal motor function. Patients with higher leptin levels showed considerable lower motor function in terms of jumping, walking and standing up from floor (D1) (p = 0.034, OR 0.976), D2 (p = 0.029, OR 0.957) and total motor function scores (p = 0.026, OR 0.973), whereas motor function in handling with objects / hand function (D3) showed no significant tendency (p = 0.103, OR 0.982).

#### Insulin and glucose

Fasting samples could only be obtained in 17/43 patients. Four patients 4/17 (23%) showed decreased and 2/12 (12%) patients increased plasma insulin concentrations. There was no association between fasting insulin and sex (p = 0.570) or SMA type (p = 0.284). Fasting glucose was determined in 15 fasting patients and was normal in 87%; only two patients had elevated glucose levels.

None of the patients in whom HbA1c was measured (41/43) showed deviations in HbA1c levels, indicating a lack of major blood glucose elevations over the preceding 3 months. In 8/15 (53%) patients HOMA index was elevated, indicating an insulin resistance; there were no associations between HOMA index and sex (p = 0.446) or SMA type (p = 0.243).

#### Urinary steroid profile

In 36/43 patients, spot morning urine samples were obtained to measure steroid excretion. Normal patterns were found in 22/36 (61%), 12/36 (33%) showed aberrant patterns with an unspecific increase of the C19 fraction (androgen metabolites) and/or the C21 fraction (cortisol metabolites). 2/36 (6%) patients showed pathological profiles with an elevation of aldosterone metabolites and mild elevations of markers for 21-hydroxylase deficiency, respectively.

### Auxological data (growth parameters)

**Fig 2:**
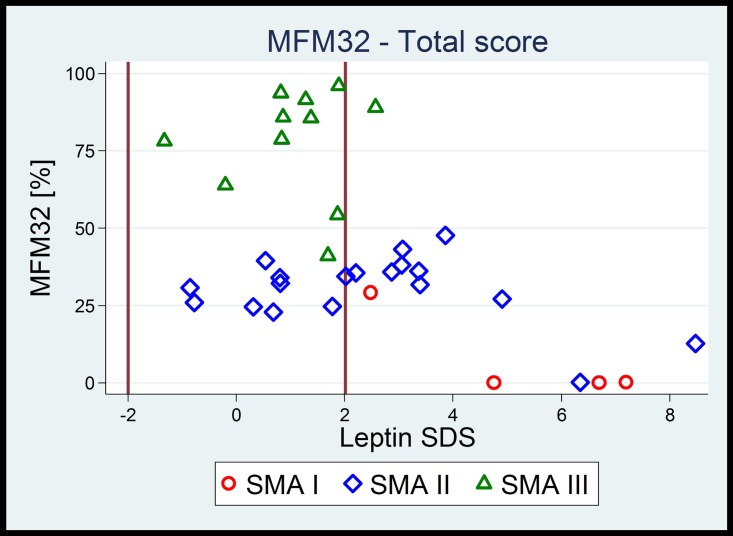
Distribution of auxological data in SMA patients (BMI = body mass index, WC = waist circumference, HC = hip circumference, WHR waist-to-hip ratio, SDS = standard deviation score). Vertical lines in bold (- 2 SD, + 2 SD) indicate the reference range. Boxes indicate the interquartile range (IQR), whiskers indicate 1.5xIQR, black dots are outliers. Asterisks indicate a significant deviation of the median from zero (p <0.01) with a shift towards higher values for WHR and leptin, as well as a shift to lower values for weight, height, BMI und HC.

Body height/length SDS of the total group ranged from -4.55 to +1.08 (mean -1.36, +/-1.47 SD); 13/43 (30%) patients were in the short range (-2.0 to—4.55). Maximum body height did not exceed +1.08 SDS (S1 Table and Fig 2); height/length SDS did not differ between sexes (p = 0.149). There was, however, a significant association with SMA type (p = 0.012); short stature was more prevalent in SMA type I and II.

Weight SDS of the total group ranged from -8.18 to +2.27 (mean -1.69, +/-2.24); 16/43 (37%) of our patients were underweight and 3/43 (7%) were overweight. There was no difference in weight between sexes (p = 0.540); however, more severely affected children (SMA type I and II) were increasingly underweight, in a single case even extremely underweight (-8.18 SD). The association of weight with SMA type is significant (p = 0.001).

Body mass index (BMI) SDS of the total group ranged from -6.77 to +2.70 (mean -1.25, +/- 2.26 SD). According to BMI SDS, 15/43 (35%) of the patients were underweight (<-2 SD), 3/43 (7%) were overweight (>2 SD); there were no differences between sexes (p = 0.767). However, we noted a significant association between SMA type (p = 0.004) and BMI. Considering the percentile range, 14/43 (33%) of SMA type I and II patients were extremely underweight and 2/43 (5%) were underweight. None of the SMA type III patients was underweight, 3/43 (7%) of all patients were overweight, 4/43 (9%) were obese.

Waist circumference (WC) SDS of all 41 patients ranged from -7.22 to +3.09 (mean -0.83, +/- 2.64). In 26/41 (63%) of patients WC was normal, 10/41 (24%) were below and 5/41 (13%) above normal standard. There were no differences between sexes (p = 0.974). Again, there was a significant association between SMA type (p = 0.015) and WC.

Waist-to-Hip Ratio (WHR) SDS of all 40 patients ranged from -1.16 to +3.39 (mean 1.30, +/- 1.09 SD); 10/25 (25%) of patients were above +2SD, none was below -2SD; there were no associations between WHR and sex (p = 0.564) or SMA type (p = 0.159).

### Pubertal development

In the prepubertal age range of girls (< 8.0 years) and boys (<9.0 years), none of the 14 boys and 4/9 girls showed pubic hair (Tanner stage PH 2). In one of these girls urinary steroid profile was normal, in 3 girls the profile showed non-specific aberrations. All these girls were hyperandrogenemic, other clinical signs of precocious puberty were lacking.

### Hirsutism

Hirsutism (Ferriman-Gallwey score ≥ 6) was present in 8/21 (38%) girls; it was seen in all SMA types (3/5 SMA type I, 3/9 SMA type II, SMA type III 2/7). No specific endocrine disease was found in these girls; 7 of the 8 girls with hirsutism had hyperleptinemia. There was no association between hirsutism and obesity or increased visceral fat tissue, defined by BMI >97. P., WHR >+2SD and WtHR >0.5.

### Motor Function Measurement Scales 32 (MFM32)

In standing position and transfers (D1), the patients reached values from 0–89% (mean 18.5%, median 2.65%), in axial and proximal motor function (D2) 0–100% (mean 54.1%, median 50%), in distal motor function (D3) 0–100% (mean 58.5%, median 57%) and a total result of 0–96% (mean 40.6%, median 34.4%) (Tab.2). There was no association between sex and motor function (p = 0.959).

## Discussion

The involvement of energy metabolism for developing dystrophy in SMA patients has not been investigated so far. In our study, hyperleptinemia was found in 15/35 (43%) of our patients; more than the half of them was underweight (BMI). Elevated leptin levels were strongly associated with lower motor function (Fig 1, Table 3) and SMA type (Tables 1 and 2); this has not been reported in SMA patients.

Hyperleptinemia increases energy expenditure, lipolysis and loss of appetite [30], thus enhancing dystrophy in SMA patients. Hyperleptinemia is associated with the SMA type although this association seems to be more an effect of the current motor function (1ure 1). Our cohort is young (mean age 8.63 years), and half of the children are prepubertal. Thus, the current motor function strongly reflects the SMA type in contrast to the later course of the disease.

In the general pediatric population, leptin levels correlated well with BMI but not with visceral fat tissue [31]. In our cohort, elevated leptin levels were not linked to increased BMI, WHR or weight. The association of high leptin levels with low motor function in the ability to walk (D1), the ability to sit upright (D2) and total score of the MFM32 might be due to the fatty remodeling of proximal and axial muscles within the progression of the disease [32, 33]. It has been hypothesized that muscle tissue, converted into white fat tissue in severely affected patients, can also produce leptin [31, 34]. The remodeling of distal muscle (hand function/D3) seems to have no influence on leptin levels due to the small amount of tissue.

In SMA patients, hyperleptinemia could also appear due to central neurodegenerative changes in the disease course. In the SMA mouse model (Smn-/-; SMN2 mice), hippocampal neurogenesis was impaired [8]. In patients with Alzheimer disease (AD), increased leptin levels in cerebrospinal fluid (CSF) and hippocampal tissue alterations were found at autopsy. However, the level of the leptin receptor mRNA was decreased in AD brain tissue so that neuronal leptin resistance was assumed to be a consequence of hippocampal alterations. Moreover, the severity of the disease positively correlated with CSF leptin concentration [35]. In SMA patients leptin levels in CSF has not been determined, so far.

With regard to weight development, we found that 3/43 (7%) of patients showed obesity measured by BMI and 10/40 (25%) by WHR, indicating an increase of visceral fat tissue as a possible sign for inhomogeneous fat distribution. Moreover, there are studies linking increased visceral fat to higher cardiovascular risk in children [20]. Therefore, measurement of WHR should be included in the standard of care program for all SMA patients.

The data for weight and height in our group of SMA I and II patients match the data reported in literature [36] with an increased occurrence of underweight and short stature.

Glucose and insulin metabolism in our group showed an increased risk for developing insulin resistance; however, HbA1c levels were normal in all our patients. In non-diabetic patients with myotonic dystrophy type 1 (DM1), another multisystemic disease, serum leptin concentration and its relation to metabolic syndrome were evaluated. It could be shown that hyperleptinemia was positively correlated with insulin resistance [37] but the clinical implications of this finding remain unclear.

Hirsutism and premature pubarche could frequently be found in our patients. Hirsutism in our patients was strongly associated with hyperleptinemia. Urinary steroid profiles showed an aberrant pattern in 14/36 (38%) of our patients, possibly as a sign for an unspecific activation of the adrenal gland. An increase in energy expenditure (hypermetabolism), amongst others, might activate the adrenal gland including clinical signs of hirsutism and premature pubarche [38]. A previous retrospective study of long-term survivors with SMA type I reported an increased occurrence of hirsutism and premature pubarche [39]. Basically, a relationship between hirsutism and obesity is not uncommon [40]. In population studies, these clinical signs were associated with an increased risk of developing polycystic ovaries, metabolic syndrome and cardiovascular problems in future life [41].

## Conclusion

1. Hyperleptinemia is associated with lower motor function and occurs more frequently in underweight SMA patients. The clinical implication could be that hyperleptinemia in SMA patients indicates a change in energy metabolism that directly leads to hypermetabolism. The necessity to start a high-energy diet in SMA patients could be guided by the measurement of leptin.
2. Leptin could also serve as a biochemical marker for disease progression. The development of hyperleptinemia in the disease course should be studied in a longitudinal multicentre study by recruiting a larger group of patients.
3. Due to the upcoming medical treatment options and the good standard of care program, SMA patients are getting older. Increased abdominal fat tissue, hirsutism and premature pubarche, which are more often seen in children with SMA type I-III, constitute risk factors for developing a metabolic syndrome. Medical follow-up of these patients from childhood up to adulthood should take this into account.

## Supporting information

S1 Table Auxiological data (SDS). (DOCX)
