# Correction: Hyperleptinemia in children with autosomal recessive spinal muscular atrophy type I-III

**DOI:** 10.1371/journal.pone.0175611

**Published:** 2017-04-06

**Authors:** Heike Kölbel, Berthold P. Hauffa, Stefan A. Wudy, Anastasios Bouikidis, Adela Della Marina, Ulrike Schara

The images for Figs. 1 and 2 are incorrectly switched. The image that appears as Fig 1 should be Fig 2, and the image that appears as Fig 2 should be Fig 1. The figure captions appear in the correct order.

**Fig 1:**
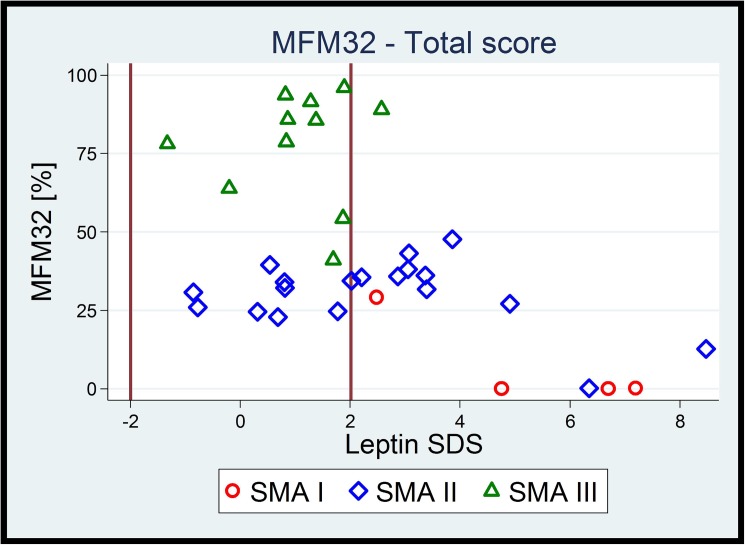
The relation between motor function and leptin SDS in terms of SMA type, showing that the lower the overall motor function, the higher was the risk for elevated leptin levels. Vertical lines in bold at -2 SD and +2 SD indicate the reference range for leptin SDS. **As a consequence, lower motor function is linked to high leptin-SDS independent of SMA type.**

**Fig 2:**
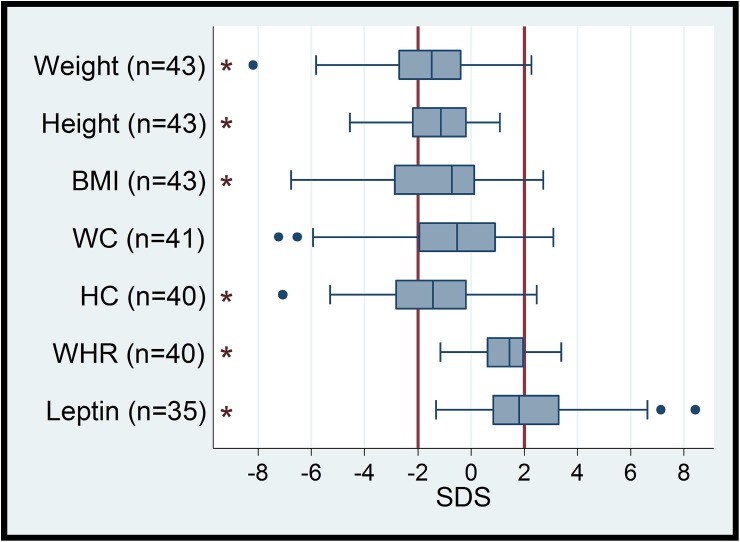
Distribution of auxological data in SMA patients (BMI = body mass index, WC = waist circumference, HC = hip circumference, WHR waist-to-hip ratio, SDS = standard deviation score). Vertical lines in bold (- 2 SD, + 2 SD) indicate the reference range. Boxes indicate the interquartile range (IQR), whiskers indicate 1.5xIQR, black dots are outliers. Asterisks indicate a significant deviation of the median from zero (p <0.01) with a shift towards higher values for WHR and leptin, as well as a shift to lower values for weight, height, BMI und HC.
