# Supplementary material for: Hyperleptinemia in children with autosomal recessive spinal muscular atrophy type I-III
Source: PLoS One. 2017 Mar 9;12(3):e0173144. doi: 10.1371/journal.pone.0173144 (PMC5344335; doi:10.1371/journal.pone.0173144)
Supplement: S1 Table — (DOCX) [file pone.0173144.s001.docx]

|  | SMA type | n | mean | SD | Median | Min-Max | < -2SD | Norm | > +2SD |
| --- | --- | --- | --- | --- | --- | --- | --- | --- | --- |
| Weight-SDS | I | 8 | -2.99 | 2.71 | -2.25 | -8.18--0.35 | 4 (50.0%) | 4 (50.0%) | 0 (0%) |
|  | II | 22 | -2.26 | 1.89 | -2.22 | -5.50-2.27 | 12 (54.5%) | 9 (40.9%) | 1 (4.5%) |
|  | III | 13 | 0.09 | 1.36 | -0.38 | -1.72-2.15 | 0 (0%) | 11 (84.6%) | 2 (15.4%) |
|  | I-III | 43 | -1.69 | 2.24 | -1.48 | -8.18-2.27 | 16 (37.2%) | 24 (55.8%) | 3 (7.0%) |
| Height/ | I | 8 | -1.76 | 1.76 | -1.83 | -4.25-1.06 | 4 (50.0%) | 4 (50.0%) | 0 (0%) |
| Length-SDS | II | 22 | -1.77 | 1.43 | -1.74 | -4.55-0.63 | 9 (40.9%) | 13 (59.1%) | 0 (0%) |
|  | III | 13 | -0.41 | 0.90 | -0.38 | -1.66-1.08 | 0 (0%) | 13 (100%) | 0 (0%) |
|  | I-III | 43 | -1.36 | 1.47 | -1.13 | -4.55-1.08 | 13 (30.2%) | 30 (69.8%) | 0 (0%) |
| BMI-SDS | I | 8 | -2.63 | 2.89 | -2.45 | -6.77-1.38 | 4 (50.0%) | 4 (50.0%) | 0 (0%) |
|  | II | 22 | -1.70 | 1.97 | -2.02 | -5.21-2.39 | 11 (50.0%) | 10 (45.5%) | 1 (4.5%) |
|  | III | 13 | 0.37 | 1.30 | 0.05 | -1.08-2.70 | 0 (0%) | 11 (84.6%) | 2 (15.4%) |
|  | I-III | 43 | -1.25 | 2.26 | -0.71 | -6.77-2.70 | 15 (34.9%) | 25 (58.1%) | 3 (7.0%) |
| WC-SDS | I | 7 | -0.79 | 2.98 | -0.52 | -7.22-1.81 | 1 (14.3%) | 6 (85.7%) | 0 (0%) |
|  | II | 21 | -1.86 | 2.61 | -1.66 | -6.53-2.74 | 9 (42.9%) | 11 (52.4%) | 1 (4.8%) |
|  | III | 13 | 0.82 | 1.63 | 0.70 | -1.33-3.09 | 0 (0%) | 9 (69.2%) | 4 (30.8%) |
|  | I-III | 41 | -0.83 | 2.64 | -0.52 | -7.22-3.09 | 10 (24.4%) | 26 (63.4%) | 5 (12.2%) |
| HC-SDS | I | 7 | -1.99 | 2.33 | -1.26 | -7.08--0.15 | 2 (28.6%) | 5 (71.4%) | 0 (0%) |
|  | II | 21 | -2.38 | 2.01 | -2.73 | -5.30-2.47 | 14 (66.7%) | 6 (28.6%) | 1 (4.8%) |
|  | III | 12 | -0.20 | 1.45 | -0.85 | -1.91-2.05 | 0 (0%) | 11 (91.7%) | 1 (8.3%) |
|  | I-III | 40 | -1.66 | 2.11 | -1.43 | -7.08-2.47 | 16 (40.0%) | 22 (55.0%) | 2 (5.0%) |
| WHR-SDS | I | 7 | 1.80 | 0.88 | 1.53 | 0.84-3.23 | 0 (0%) | 5 (71.4%) | 2 (28.6%) |
|  | II | 21 | 1.00 | 1.08 | 0.97 | -1.16-2.71 | 0 (0%) | 18 (85.7%) | 3 (14.3%) |
|  | III | 12 | 1.51 | 1.14 | 1.47 | -0.43-3.39 | 0 (0%) | 7 (58.3%) | 5 (41.7%) |
|  | I-III | 40 | 1.30 | 1.09 | 1.44 | -1.16-3.39 | 0 (0%) | 30 (75.0%) | 10 (25.0%) |
| Table 4: Distribution of auxological data (SDS) in SMA types I-III | | | | | | | | | |
